# Stand-Alone Mobile Mindfulness App for People Experiencing Infertility: A Pilot Randomised Controlled Trial (MoMiFer-RCT)

**DOI:** 10.5334/pb.1375

**Published:** 2025-10-06

**Authors:** Tessy Boedt, Nele Willaert, Sharon Lie Fong, Eline Dancet, Filip Raes, Katleen Van der Gucht

**Affiliations:** 1 Department of Chronic Diseases and Metabolism, KU Leuven, Leuven, Belgium; 2 Tilburg School of Social and Behavioral Sciences, Tilburg University, Tilburg, The Netherlands; 3 Faculty of Psychology and Educational Sciences, KU Leuven, Leuven, Belgium; 4 Leuven University Fertility Centre, University Hospitals Leuven, Leuven, Belgium; 5 Department of Development and Regeneration, KU Leuven, Leuven, Belgium; 6 Department of Rehabilitation Sciences, Neuromodulation Laboratory, Biomedical Sciences Group, KU Leuven, Leuven, Belgium

**Keywords:** infertility, emotional distress, quality of life, mindfulness, mobile mindfulness app

## Abstract

**Objectives:**

Infertility and its treatments result in considerable emotional burden. This study aimed to examine the impact of a stand-alone mobile mindfulness application (MoMiFer-app).

**Methods:**

A pilot RCT was conducted with participants randomized into an intervention group (n = 34) using the MoMiFer-app, and a wait-list control group (n=38). Outcomes were collected at baseline, 1.5 months, and 3 months post-randomization using experience sampling method (ESM) and self-report questionnaires. Primary outcomes included symptoms of emotional distress (DASS-21+ESM) and fertility-related quality of life (FertiQoL). Secondary outcomes assessed repetitive negative thinking (PTQ), self-compassion (SCS-SF), and mindfulness skills (CHIME-SF+ESM). App usage was evaluated through app-tracking.

**Results:**

Multilevel analysis showed no significant improvement in primary outcomes. However, the MoMiFer-app significantly enhanced self-compassion and mindfulness skills, as assessed by self-report questionnaires. A significant condition×time effect was observed for mindfulness skills at 1.5 months (T1; p = .02) and 3 months (T2; p = .02), and for self-compassion at 3 months (T2; p = .006). No effect was observed on repetitive negative thinking. The app was rated as good quality, but nearly half of the participants (47%) practiced mindfulness with the app once a week or less.

**Conclusions:**

Online mindfulness-based interventions can be valuable in fertility care providing easily accessible low-intensive mental support, even if they do not directly improve emotional distress or quality of life in the short term. The trial’s timing during the COVID-19 pandemic and low app usage may have influenced outcomes. Further research on potential stressors and ways to increase user adherence is needed to better understand the app’s impact.

## Introduction

Infertility and its treatments (Zegers-Hochschild et al., 2017) result in considerable emotional burden for women, men, and couples (Luk & Loke, 2015; Verhaak et al., 2005, 2007). Studies indicate that approximately 30–60% of women and 10–30% of men experience emotional burden during this period (Agostini et al., 2017; Chachamovich et al., 2010; Huppelschoten et al., 2013; Milazzo et al., 2016; Nik Hazlina et al., 2022; Rooney & Domar, 2018). This emotional burden often includes feelings of grief, loss and frustration and can lead to anxiety, depression, and a diminished sense of self-worth and quality of life (Patel et al., 2018; Rooney & Domar, 2018). Moreover, several studies have shown that this emotional burden may be an important reason for couples to terminate assisted reproductive technology interventions without achieving a live birth (Domar et al., 2018; Gameiro et al., 2012).

Different supportive psychosocial interventions have been developed for people with infertility across the treatment cycle (Ying, Wu & Loke, 2015), including Mindfulness Based Interventions (MBIs) targeting women with infertility (Dalal, 2023; Wang, Liu & Lei, 2023). The main goal of MBIs is to actively train people to increase awareness of one’s present-moment experience (thoughts, emotions, bodily sensations) with an accepting, open, and non-judgmental attitude (Crane et al., 2017; Kabat-Zinn, 2014). A recent systematic review and meta-analysis that included only randomised controlled trials (RCT), focused on the effects of face-to-face MBIs for women with infertility (Wang, Liu & Lei, 2023). Large, significant effects for anxiety and depression were found, with these effects being maintained for up to 3 months post-intervention. Significant improvements were also observed in several domains of quality of life, although no effect was found on perceived stress. Mindfulness skills seemed to be enhanced through MBIs as well (Galhardo, Cunha & Pinto-Gouveia, 2013, 2019). Importantly, the body of research on the effectiveness of MBIs for infertile men and couples is more limited (Dalal, 2023; Gaitzsch et al., 2020).

Research regarding the effectiveness of online and smartphone-delivered self-administered interventions as a tool to support mental health is growing (Gál, Ștefan & Cristea, 2021; Jayewardene et al., 2017; Linardon et al., 2019; Spijkerman, Pots & Bohlmeijer, 2016). What is key here, is that such tools make low-intensive psychological help easily accessible for a large audience at low-cost. A recent meta-analysis found promising results for mindfulness meditation apps in clinical and non-clinical populations for multiple psychological outcomes including anxiety, depressive symptoms, quality of life, and perceived stress (Gál, Ștefan & Cristea, 2021). However, research data in this area are lacking for people with infertility.

Given the emotional burden of infertility and its treatment (Luk & Loke, 2015, Van Dongen et al., 2012; Verhaak et al., 2005, 2007), and the time cost of fertility treatment, people experiencing infertility require easily accessible and low intensive strategies to improve their mental health (Boedt et al., 2021). Therefore, the overall goal of this study was to examine the effect of a stand-alone mobile mindfulness app (MoMiFer-app) vs. wait-list control on symptoms of emotional distress (symptoms of stress, anxiety, and depression) and fertility-related quality of life in people experiencing infertility. We hypothesized a reduction in symptoms of emotional distress and an increase in fertility-related quality of life in people using our MoMiFer-app. Secondly, we aimed to explore underlying mechanisms of change of MBI’s (Alsubaie et al., 2017). Specifically, we hypothesized that changes in symptoms of emotional distress were associated with improvements in mindfulness skills and self-compassion on the one hand, and a reduction in repetitive negative thinking on the other hand.

To examine these hypotheses, we combined self-report questionnaires (on emotional distress, fertility related quality of life, mindfulness skills, self-compassion and repetitive negative thinking) and the experience sampling method (ESM). ESM is regarded as an ecologically valid method for capturing in-the-moment assessments of people’s daily experiences, while minimizing retrospective bias (Csikszentmihalyi & Larson, 2014). The current study employed ESM to investigate in-the-moment emotional distress and mindfulness skills in participants’ daily life.

## Method

The MoMiFer-research project was a pilot randomised controlled trial (RCT) (Hallingberg et al., 2018) in which the CONSORT guidelines (Schulz, Altman & Moher, 2010) were followed. A detailed protocol of the study has been registered (ClinicalTrials.gov Identifier: NCT04143828) and published previously (Boedt et al., 2022).

### Ethics and consent

This study was approved by the Ethics Committee Research UZ/KU Leuven (Belgium) (approval number: S62323). Any subsequent protocol amendments were submitted to the appropriate Ethics Committee and National Regulatory Authorities for approval. Participants IDs were used to guarantee confidentiality of participant’s data (i.e., coding of dataset). All participants provided written informed consent after receiving detailed information about the study procedures, confidentiality, and data protection measures. Participants were informed that their involvement was voluntary and that they could withdraw from the study at any time without justification.

### Eligibility criteria

Inclusion criteria were people, including women and their partners, experiencing infertility (Zegers-Hochschild et al., 2017), speaking and understanding Dutch, aged 18–43, and who were in possession of a smartphone. Participants who were receiving fertility treatment and/or utilizing concomitant care, such as acupuncture or fertility counselling, were considered eligible for inclusion in the study. This information was assessed at baseline and the end of the study.

### Recruitment and study setting

Recruitment through open enrolment ran from November 24^th^, 2019 through July 5^th^, 2021. Initially, participants were recruited through two Belgian non-profit organisations for people experiencing infertility ‘De Verdwaalde Ooievaar’ (https://www.deverdwaaldeooievaar.be/) and ‘Kinderwens’ (https://www.kinderwens.org/). Both organisations, together with ‘PraxisP’, the academic practice centre of the University of Leuven, promoted the study via their online channels. Promoting the study through social media accounts (e.g., Instagram) and the distribution of flyers at the fertility centre of the University Hospitals Leuven provided additional ways to reach the targeted sample size.

### Randomisation, blinding and treatment allocation

A computer-automated randomisation procedure was applied via a password-protected website (www.uzleuven.be/rct), using randomly varying block sizes and a 1:1 allocation ratio. This procedure was performed by one of the members of the researcher team. Due to the study design, the participants were not blinded for treatment allocation. Participating couples were randomly assigned as a single unit. The woman and her partner were considered as two individual participants. A personal code was assigned to each participant. The investigators who analysed the data could identify participants only by this code, not by their names.

### Procedure

A member of the research team mailed the informed consent to potential participants. Consenting participants were invited for study intake through an online video call. During this standard intake additional questions regarding the study were answered and the assessment methods were explained. Once consent was given, participants completed the baseline measurements (T0). An overview of outcomes, methods for data collection, and timing of data collection is displayed in Table 1. After baseline assessment, randomisation took place as described above. Next, the MoMiFer-app was installed, providing the complete stand-alone mobile mindfulness application (experimental group) or the version with solely information regarding the study (wait-list control group). When assigned to the control group, participants gained access to the complete MoMiFer-app after 3 months. Participants received an overview of individualised assessment moments. Reminders via the app and email were sent one week and one day before the 1.5 month and 3 month follow-up assessments, to promote participant retention and complete follow-up measurements. If a clinical pregnancy (Zegers-Hochschild et al., 2017) occurred during the trial, participation in the study was terminated.

**Table 1:** Outcomes, method(s) of assessment and timing of assessments. Q, questionnaire; ESM, experience sampling method.


OUTCOMES	METHOD(S) OF ASSESSMENT	TIMING OF ASSESSMENT		

		BASELINE	1.5 MONTHS	3 MONTHS

**Primary outcome measures**				

Quality of life	Self-report Q	X	X	X

Emotional distress	ESM	X	X	X

	Self-report Q	X	X	X

**Secondary outcome measures**				

Mindfulness skills	Self-report Q	X	X	X

	ESM	X	X	X

Repetitive negative thinking	Self-report Q	X	X	X

Self-compassion	Self-report Q	X	X	X

User-rated quality of the app	Self-report Q			X

Use of the app	App-tracking	X	X	X

Background information	Self-report Q	X		


### Intervention

**Figure 1:**
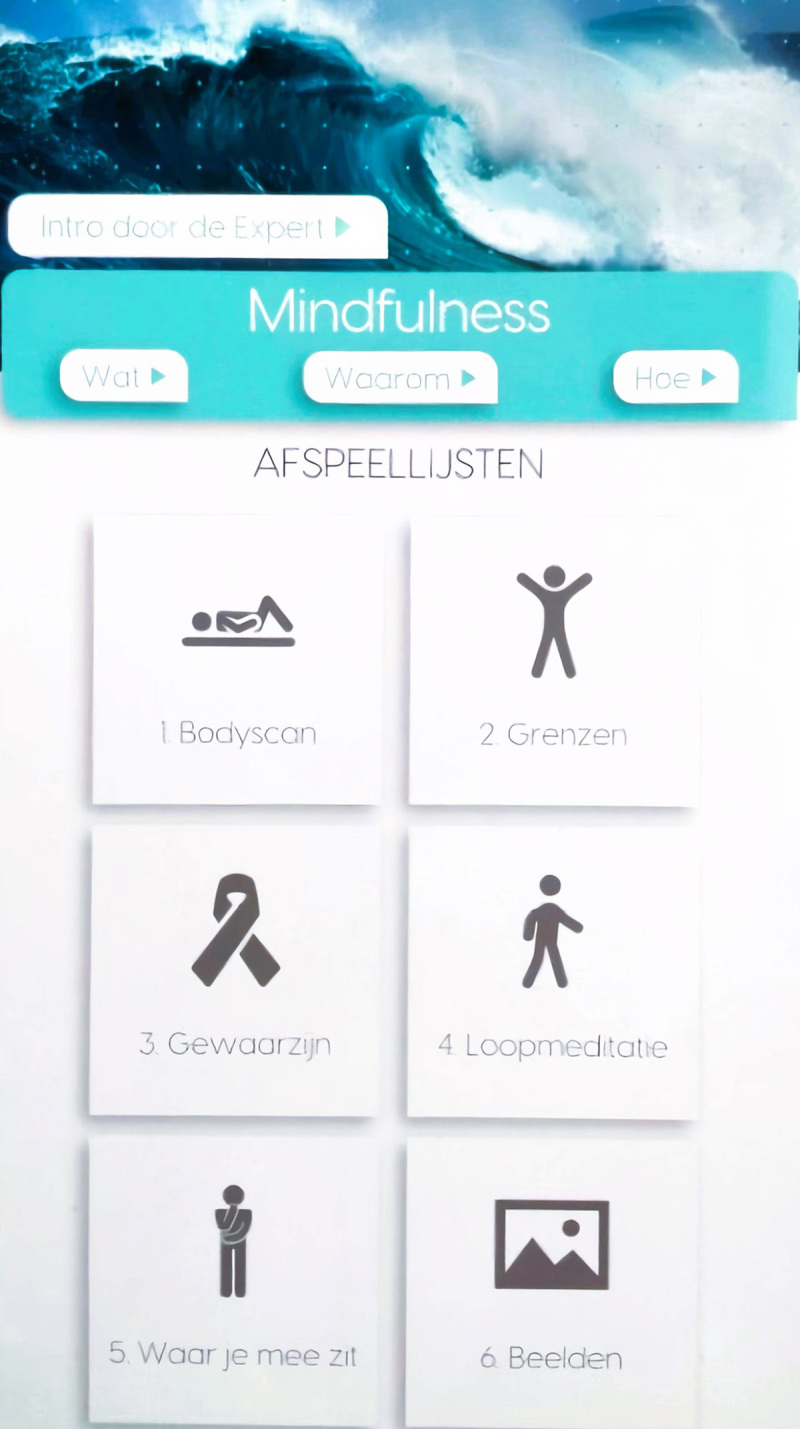
App interface showing the six consecutive mindfulness modules.

A patient-centred design was applied for creating the MoMiFer-app by involving both patients and healthcare professionals in the development process (Boedt et al., 2019; Craig et al., 2008; MRC, 2000). The MoMiFer-app included mindfulness exercises following the format and content of Mindfulness-Based Stress Reduction (Crane et al., 2017; Kabat-Zinn, 2014; Maex, 2006). The exercises were spread over six consecutive modules, including practices such as the body scan, walking meditation, and sitting meditation with a focus on breathing awareness. An image of the app interface is shown in Figure 1. Each module consisted of a short educational video clip (talking head) explaining the content, and two audio files (between three and 45 minutes) with experiential mindfulness meditation exercises. Participants could follow the different modules based on their own schedules over the 3-month study period. Additionally, participants had access to answers to frequently asked questions on mindfulness and fertility in the MoMiFer-app.

### Sample size

Sample size was based on the experience sample method (ESM) applying the 30/30 rule, used to determine sample size in multilevel modelling, which recommends sampling 30 participants with 30 observations per group (Hox, Moerbeek & van de Schoot, 2010). This sample size is known to achieve a sufficient statistical power to detect a moderate-to-large effect size for a single fixed effect (Mathieu et al., 2012; Scherbaum & Ferreter, 2009). The effect was the change in symptoms of emotional distress as measured by ESM. The same approach was used in previous related research (Van der Gucht et al., 2019).

### Outcomes

Socio-demographic characteristics, fertility-related information (i.e., fertility treatment, infertility diagnosis, and causes of infertility), concomitant care, and prior experience with mindfulness were reported by the participants during baseline measurements. An overview of outcomes, methods for data collection, and timing of data collection is displayed in Table 1.

#### Primary outcomes

Primary outcomes were fertility-related quality of life and symptoms of emotional distress. Fertility-related quality of life was surveyed with the Fertility Quality of Life Questionnaire (FertiQoL) (Aarts et al., 2011; Boivin, Takefman & Braverman, 2011). The Core FertiQoL captures the average fertility quality of life across four domains i.e. the emotional, relational, mind-body, and social domain. The items are rated on a 4-point Likert scale (0–4) using different response categories. Total scores are summed and scaled to a 0–100 scale, with higher scores indicating better fertility-related quality of life.

Symptoms of emotional distress were measured with the Depression, Anxiety, and Stress Scale-21 (DASS-21) (de Beurs et al., 2001), and three ESM-items on depression, anxiety and stress (Csikszentmihalyi & Larson, 2014, pp. 35–54). The DASS-21 is the shortened version of the original DASS (de Beurs et al., 2001). Responses are rated on a 4-point Likert scale (0 = *did not apply to me at all*, to 3 = *applied to me very much most of the time*). The total score is calculated through summing the item scores and multiplying the result by two, to align with the original DASS scoring system (0–126). Higher scores are associated with greater symptoms of emotional distress.

ESM is a validated, structured diary technique to assess participants in the context of their daily living environment (Csikszentmihalyi & Larson, 2014, pp. 35–54). During assessment, participants’ smartphones beeped 10 times/day for 4 consecutive days according to a semi-stratified interval scheme (waking hours were divided into 10 equal intervals and in each interval one beep was randomly programmed). Participants received the same set of questions at each beep and had a 10-minute window to respond before the notification expired. The ESM items were based on previous research (see supplementary material) (Van der Gucht et al., 2019). To assess symptoms of emotional distress, three ESM questions (one each for depression, anxiety, and stress) were administered via the app at each beep using a sliding scale ranging from 0 to 100 (0 = *not at all* to 100 = *very much or extremely*). A mean score across the three items was computed for each assessment phase by averaging responses over the four-day period resulting in scores ranging from 0 to 100. Higher scores indicated more symptoms of emotional distress.

#### Secondary outcomes

Secondary outcomes included mindfulness skills, repetitive negative thinking, self-compassion, the user-rated quality of the app, and use of the app. Mindfulness skills were measured with ESM (mindfulness-state) and the Comprehensive Inventory of Mindfulness Experiences-Short Form (mindfulness trait) (CHIME-SF; Cladder-Micus et al., 2019). Seven ESM items questioned aspects of mindfulness in daily life via a sliding scale in the app ranging from 0 to 100 (supplementary material). Similar to the scoring method of the ESM items on emotional distress, a mean score across the four days was calculated for each assessment phase. Higher ESM scores indicated better mindfulness skills. The self-report CHIME-SF questionnaire measures a broad range of mindfulness aspects (Cladder-Micus et al., 2019). The short form comprises 24 items rated on a 6-point Likert scale (1 = *almost never* to 6 = *almost always*). The total score is calculated by summing all item responses, resulting in a possible range of 0 to 144, with higher scores reflecting greater mindfulness skills.

Repetitive negative thinking was assessed with the Perseverative Thinking Questionnaire (PTQ; Ehring et al., 2011, 2012). The PTQ contains the following subscales: core features of RNT (repetitiveness, intrusiveness, and difficulty disengaging), perceived unproductiveness of RNT, and the extent to which RNT occupies mental capacity. Participants were asked to rate each item on a 5-point Likert scale (0 = *never* to 4 = *almost always*). The total score was calculated by summing all item responses, yielding a possible range from 0 to 60, with higher scores reflecting greater levels of RNT.

The Self-Compassion Scale-Short Form (SCS-SF; Neff, 2016; Raes et al., 2011) was used to assess self-compassion. This self-report questionnaire consists of 12 items covering six subscales: self-kindness, common-humanity, mindfulness, self-judgment, isolation, and over-identification. The items are rated on a 5-point Likert scale (1 = *almost never* to 5 = *almost always*). A mean score is calculated across all items, resulting in a total score between 1 and 5, with higher scores indicating more self-compassion.

In the experimental group, the user-rated quality of the app was obtained at the end of the study using the short version of the subjective quality subscale from the MARS (Stoyanov et al., 2015). This included two items: one on app recommendation (“Would you recommend this app to people who might benefit from it?”) and one on overall rating (“What is your overall star rating of the app?”). Each item was scored on a 5-point Likert scale. The total score, ranging from 0 to 8, was calculated by summing the scores of both items, with higher scores suggesting greater perceived app quality. Use of the MoMiFer-app was monitored through app tracking. We tracked how often participants in the experimental group opened the application and which exercises they performed.

### Data management

Self-report questionnaire data were collected using Qualtrics (www.qualtrics.com). ESM data were collected in the MoMiFer-app (Csikszentmihalyi & Larson, 2014, pp. 35–54). Storage and analysis were done by the study investigator in SPSS according to good clinical practice. Deviation of maximally 1 week before and after the planned time point was allowed. The data from the mobile mindfulness application could be retrieved from the secured website of MoMiFer to which only the research team had access.

### Data analysis

The minimal required compliance was set at 30%, a common directive in ESM research (Kramer et al., 2014). Hierarchical linear modelling (HLM) was applied to examine differential trajectories (Raudenbush & Bryk, 2001). Estimator for missing data was full information maximum likelihood. To test the experimental effect, a multilevel model was used with two levels: time-points (Level-1) were nested within persons (Level-2). Time-points were represented by a dummy variable, with time coded as baseline (T0), 1.5 month follow-up (T1), and 3 month follow-up (T2). The same multilevel model with a random intercept was applied to each outcome. In this model, (a) the dummy-coded assessment time (as a level-1 variable), (b) the treatment condition (as a level-2 variable), and (c) their cross-level interactions were included in predicting the outcome. The analysis was performed in R (https://www.r-project.org/), with use of the ‘lme4’ package (Bates et al., 2015). The Cohen’s *d* statistic was calculated to compute within- and between-group effect sizes. A *p* < .05 corrected for multiple testing (Benjamini & Hochberg, 1995), determined statistical significance for the experimental group.

### COVID-19 pandemic and its impact on the fertility trajectory

During recruitment the COVID-19 pandemic began (Federal Public Services [FPS], 2020). The pandemic limited, amongst others, access to fertility treatment. A broad exploratory open-ended research question was added at each assessment phase through mailing the participants, to gain insight in the influence of the pandemic on participants’ fertility trajectory.

Thematic data analysis, as outlined by Braun and Clarke (2006) was applied to process and analyse the responses of the participants. An overall description of the data set was provided in order to obtain a sense of the important themes, using an inductive approach. Prevalence of a theme was defined by counting the participants who mentioned it. One answer could fit different themes, yielding more than one count.

## Results

### Enrolment and attrition

**Figure 2:**
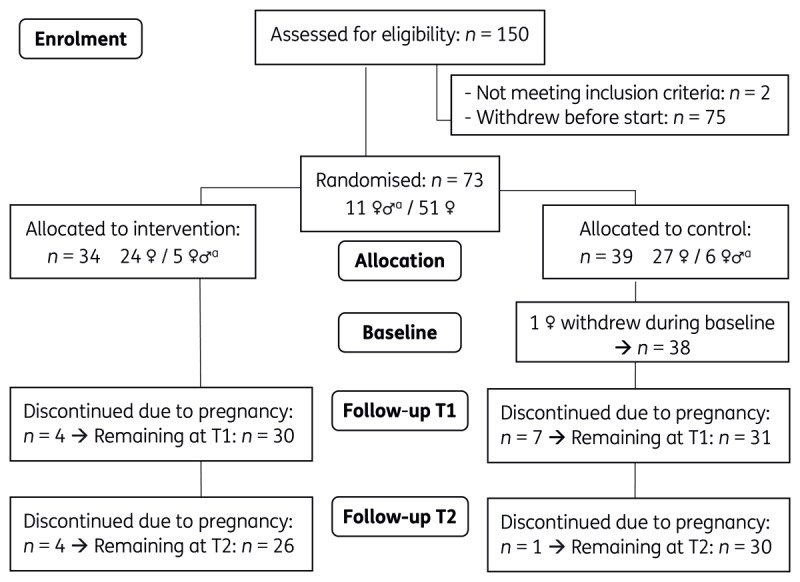
CONSORT flowchart of participants. *Note*. T1 = 1.5 months after randomisation; T2 = 3 months after randomisation. ^a^ The ♀♂ symbol denotes the total number of couples participating in the study, with each couple counted as two individual participants.

The CONSORT flowchart (http://www.consort-statement.org/) shows enrolment and attrition of participants (Figure 2). Eligibility was assessed for 150 people. Six eligible participants were unable to participate because of pregnancy before the start of the study. Two women who withdrew before the study began, cited the time investment and demanding nature of the study as reasons for not participating. In total, 73 participants were randomised. One woman withdrew after randomisation, because she was assigned to the control group and indicated the need for immediate mental support. Couples participating in the study were allocated to the same group and were each considered as an independent participant. Specifically, a total of 11 heterosexual couples participated, counting for 22 participants. Three couples and 10 women discontinued during the study due to a clinical pregnancy (Zegers-Hochschild et al., 2017). Given the low number of couples, and hence men, data analysis was solely performed at the individual level.

### Descriptive statistics

Table 2 summarizes the demographic characteristics at baseline for both groups. For most participants, the highest level of education obtained was a college degree (35%) or a university master’s degree (47%). Most of the participants were currently going through fertility treatment (81%), or followed treatment in the past (88%). Women made relatively greater use of concomitant care (69%) than men (18%). Most participants (*n =* 54, 75%) had no prior experience with mindfulness. The primary and secondary outcome data for the three assessment phases are presented in Tables 3 and 4, respectively. The attrition rate for the Experience Sampling Method (ESM) was higher than for the retrospective self-report questionnaires. Specifically, overall compliance with ESM was 65% across the three assessment phases (T0 = 82%, T1 = 49%, T2 = 50%), whereas compliance with the self-report questionnaires was 83% (T0 = 99%, T1 = 80%, T2 = 71%).

**Table 2:** Sociodemographic Characteristics of Participants at Baseline.


Baseline characteristic	*n* (%)		*p*

	CONTROL	INTERVENTION	

Age: *M* ± *SD*	32.3 ± 3.2	31.3 ± 3.9	.23

Sex: ♂/♀	6 (16)/32 (84)	5 (15)/29 (85)	.9

Employment status			.8

Full time^a^	29 (76)	27 (79)	

Parttime^a^	6 (16)	6 (18)	

Unemployed, deliberately unemployed, incapacitated^a^	3 (8)	1 (3)	


*Note*. *M* = mean; *SD* = standard deviation. ^a^ Reflects the number and percentage of participants answering ‘yes’ to this question.

**Table 3:** Descriptive Statistics of the Primary Outcomes.


					SYMPTOMS OF EMOTIONAL DISTRESS					

		FERTIQOL			DASS-21			ESM		

		WAVE								

Group	Statistic	T0	T1	T2	T0	T1	T2	T0	T1	T2

C	*M*	56.9	42.4	58.9	40.8	40.8	39.1	19.1	20	16.7

	*SD*	13.3	12.8	11.8	19.4	21.7	23.6	9.1	15.5	9.1

	*n*	34	23	18	38	24	20	28	12	15

	Missing	4	8	12	0	7	10	10	19	15

I	*M*	59.4	46.4	63.1	38.3	36.2	25.2	21.5	19	19.7

	*SD*	13.2	9.9	12.9	20.4	20.7	15.1	13	14.3	13.8

	*n*	33	24	20	34	25	20	31	18	13

	Missing	1	6	6	0	5	6	3	12	13


*Note*. FertiQoL = fertility related quality of life; DASS-21 = depression, anxiety, and stress scale – 21 items; ESM = experience sampling method; T0 = baseline; T1 = 1.5 months after randomisation; T2 = 3 months after randomisation; C = control; I = intervention.

**Table 4:** Descriptive Statistics of the Secondary Outcomes.


					MINDFULNESS SKILLS								

		PTQ			CHIME-SF			ESM			SCS-SF		

		WAVE											

GROUP	STATISTIC	T0	T1	T2	T0	T1	T2	T0	T1	T2	T0	T1	T2

C	*M*	34.5	33	32.2	83	80	79	57	58.9	62	2.7	2.6	2.6

	*SD*	10.5	9.7	8.4	14	11	11	10.2	7.8	10.8	.7	.7	.6

	*n*	38	24	20	38	24	20	28	12	15	38	24	20

	Missing	0	7	10	0	7	10	10	19	15	0	7	10

I	*M*	32	31	26	83	87	90	56.6	61.7	61.4	2.9*	3.1	3.3*

	*SD*	11	9	10	13	13	14	13	11.8	11.3	.6	.7	.7

	*n*	34	24	20	34	24	20	31	18	13	34	24	20

	Missing	0	6	6	0	6	6	3	12	13	0	6	6


*Note*. PTQ = perseverative thinking questionnaire; CHIME = comprehensive inventory of mindfulness experiences; ESM = experience sampling method; SCS-SF = self-compassion scale – short form; T0 = baseline; T1 = 1.5 months after randomisation; T2 = 3 months after randomisation; C = control; I = intervention. *p < .05.

### Intervention effect on primary outcomes based on multilevel analysis

Multilevel analyses revealed that no significant condition × time effects were observed for symptoms of emotional distress, and fertility-related quality of life and time (see Table 5). Thus, at both time points (1.5 months and 3 months), no significant decrease in symptoms of emotional distress nor a significant increase in fertility-related quality of life was observed for the intervention group compared with the control group.

**Table 5:** Results of HLM Estimating the Intervention and Time effect on Primary Outcomes.


OUTCOME	FACTORS										

	T0	T1		T2		I		T1 × I		T2 × I	

	*β_0_* ± *SE*	*β* ± *SE*	*p* ^a^	*β* ± *SE*	*p* ^a^	*β* ± *SE*	*p* ^a^	*β* ± *SE*	*p* ^a^	*β* ± *SE*	*p* ^a^

FertiQoL	57.08 ± 2.1	–14.52 ± 1.5	<.001	2.01 ± 1.9	.31	2.29 ± 3.1	.46	1.19 ± 2.1	.56	1.17 ± 2.7	.66

DASS-21	40.79 ± 3.3	0.70 ± 3.9	.86	–3.12 ± 4.2	.46	–2.50 ± 4.8	.61	–1.78 ± 5.5	.75	–7.87 ± 6	.19

ESM	19.07 ± 2.2	0.71 ± 3	.81	–1.75 ± 2.7	.52	2.39 ± 3.1	.44	–4.19 ± 3.8	.28	–1.55 ± 3.9	.69


*Note*. FertiQoL = fertility quality of life; DASS-21 = depression, anxiety, and stress scale – 21 items; ESM = experience sampling method; T1 = 1.5 months after randomisation; T2 = 3 months after randomisation; I = intervention group. ^a^ Satterthwaite’s method for t-tests.

### Intervention effect on secondary outcomes based on multilevel analysis

The results of the multilevel analysis for the secondary outcomes are displayed in Table 6. Concerning mindfulness skills, a significant condition × time effect was observed at 1.5 months (T1; *p* = .02) and 3 months (T2; *p* = .02). The effect at both time points was positive, which suggests that participants in the mindfulness group, unlike those from the control group, experienced an increase in mindfulness skills 1.5 and 3 months after using the app. In both groups no significant effect of time was found. The condition × time interaction was significant for self-compassion (*p* = .006) at 3 months (T2). Here, an increase in self-compassion was observed for the intervention group compared to the control group. In both groups no significant effect of time was found. No significant condition × time effects were observed for mindfulness skills as measured with ESM, and repetitive negative thinking.

**Table 6:** Results of HLM Estimating the Intervention and Time effect on Secondary Outcomes.


OUTCOME	FACTORS										

	T0	T1		T2		I		T1 × I		T2 × I	

	*β_0_* ± *SD*	*β* ± *SD*	*p* ^a^	*β* ± *SD*	*p* ^a^	*β* ± *SD*	*p* ^a^	*β* ± *SD*	*p* ^a^	*β* ± *SD*	*p* ^a^

PTQ	34.45 ± 1.7	–1.55 ± 1.6	.34	–3.68 ± 2.4	.13	–2.33 ± 2.4	.34	–0.21 ± 2.3	.93	–2.39 ± 3.4	.48

mindfulness ESM	57.23 ± 2.1	1.43 ± 2.5	.57	3.55 ± 2.4	.14	–0.67 ± 3	.82	5.33 ± 3.3	.11	3.73 ± 3.4	.28

mindfulness CHIME	126.82 ± 3	–2.62 ± 2.5	.31	–4 ± 3.4	.25	0.74 ± 4.4	.87	8.80 ± 3.6	.02*	11.68 ± 4.8	.02*

SCS-SF	2.69 ± 0.1	0.01 ± 0.1	.94	–0.04 ± 0.1	.76	0.16 ± 0.2	.33	0.24 ± 0.1	.08	0.49 ± 0.2	.006**


*Note*. PTQ = perseverative thinking questionnaire; ESM = experience sampling method; CHIME = comprehensive inventory of mindfulness experiences; SCS-SF = self-compassion scale – short form; T1 = 1.5 months after randomisation; T2 = 3 months after randomisation; I = intervention group. ^a^ Satterthwaite’s method for t-tests. *p < .05. **p < .01.

### Acceptability and use of the app

The subjective-app quality was questioned at the end of the study via the shortened version of the subjective quality subscale of the MARS (Stoyanov et al., 2015). Most people in the intervention group gave the app an average score (*n* = 8, 40%) or higher (*n* = 8, 40%). Four people graded the app below average (20%). At the end of the study, three participants in the intervention group indicated they had used a different mindfulness apps during the trial.

Regarding the actual use of the app, app-based tracking showed that almost half of the participants in the intervention group (*n* = 16, 47%) practiced mindfulness through the app less than once a week. Ten participants practiced once a week, and four participants practiced mindfulness at least two times a week. Practice frequency over the three-month trial period is provided in the supplementary material.

### The COVID-19 pandemic and its impact on the fertility trajectory

Most participants took part in the study during the pandemic (T0: 83%, T1: 90%, T2: 96%). However, due to a limited number of responses to the open-ended question about the pandemic’s effect on fertility (See supplementary material), results should be interpreted cautiously.

At baseline, 70% reported postponed or interrupted fertility treatment due to the corona pandemic. Nearly half of the respondents (41%) indicated the pandemic and its attendant measures led to a lack of perspective and a greater sense of unpredictability to the already highly structured and time-sensitive nature of fertility treatment. Furthermore, across the different assessment phases, 31% of respondents stated reduced partner involvement due to restrictions on attending hospital appointments. This is illustrated by the following quote: “*You are never allowed to attend an appointment in pairs [with your partner]… My husband feels like he is missing information, and I don’t always feel properly supported by him… It is very strange to feel lonely in a shared desire [to have children]*” (participant ID: FMMFV65).

## Discussion

This pilot RCT aimed to assess the impact of a stand-alone mobile mindfulness app for people experiencing infertility (MoMiFer-app) on symptoms of emotional distress and fertility-related quality of life (primary outcomes), together with the effects on potential underlying mechanisms, such as repetitive negative thinking, self-compassion and mindfulness skills (secondary outcomes).

Our findings revealed that providing the MoMiFer-app to people experiencing infertility did not yield a significant effect on the primary outcomes including symptoms of emotional distress and fertility-related quality of life. In contrast to our findings, a meta-analysis of RCTs on the efficacy of mindfulness meditation apps reported improvement in several mental health outcomes, including perceived stress, symptoms of depression and anxiety, and quality of life (Gál, Ștefan & Cristea, 2021). However, the studies encompassed in this meta-analysis did not target people with infertility. Additionally, a recent systematic review and meta-analysis evaluated the effects of MBIs for women with infertility (Wang, Liu & Lei, 2023). Although the majority of MBIs applied a face-to-face format, one study implemented an MBI delivered via an audio compact disc (Stefanaki et al., 2015). The results indicated that MBIs were effective in reducing symptoms of depressions and anxiety, while improving multiple domains of health-related quality of life (Wang, Liu & Lei, 2023).

There are several potential reasons for the lack of significant effects on the primary outcomes in our study. First, the absence of a significant effect of the MoMiFer-app on fertility related quality of life, symptoms of emotional distress could be associated with the trial’s timing, which coincided with the onset of the COVID-19 pandemic and lockdown period. A study by Boivin et al. (2020) conducted during the beginning of the pandemic, showed that women displayed heightened feelings of uncontrollability regarding their ability to achieve their goal of becoming parents. The participants reported experiencing stress, worry, and frustration. Considering these findings, it is possible that the challenging circumstances during the MoMiFer-trial have outweighed the potential positive effects of the mindfulness app. These findings are in line with another study that we carried out during the same period in a population of adolescents who suffered from a chronic condition. In this study, where we used a mixed-method approach with quantitative and qualitative data, participants mentioned that the MBI acted as a buffer against the increasing stress and worry caused by the COVID-19 pandemic (Kock et al., 2021). A replication study would be useful to eliminate the potential influence of the COVID-19 pandemic on the outcomes.

Second, it is possible that the use of the app by individuals was too low, which may have contributed to the non-significant outcome effects. In the current trial, almost half of participants practiced mindfulness through the app less than once a week, while research indicates that regular practice is recommended to have an impact (Carmody & Baer, 2008; Krusche, Cyhlarova & Williams, 2013; Parsons et al., 2017; Strohmaier, 2020).

Additionally, the current study focused on a stand-alone mobile mindfulness intervention without any reminders or motivational tools, based on patient preferences (Boedt et al., 2021). However, a meta-analysis of smartphone apps teaching mindfulness, acceptance, and self-compassion skills found that studies including reminders to use the app showed significantly larger effect sizes compared to those without reminders (Linardon, 2020). This aligns with the findings of Lippmann et al. (2019), who identified timely information in the form of reminders (e.g., via email) as a key element of effective internet-based mindfulness interventions to encourage consistent practice. Future research should therefore aim to balance patient preferences (i.e., minimal reminders) with the need for reminders to ensure effective interventions. Furthermore, exploring what enhances overall user adherence, could make online mindfulness practice more accessible and effective. (Linardon, 2020; Carmody & Baer, 2008; Krusche, Cyhlarova & Williams, 2013; Parsons et al., 2017; Strohmaier, 2020).

In terms of the secondary outcomes, the study showed a significant positive impact of the MoMiFer-app on self-compassion and mindfulness skills, as assessed by self-report questionnaires. However, no significant impact was observed on mindfulness skills measured by ESM.

An increase in self-compassion and mindfulness skills may indirectly benefit patients coping with infertility-related stress as it can serve as an emotional regulation strategy against feelings of self-criticism for infertility (Raque-Bogdan et al., 2015). This suggests that mindfulness-based interventions can be a valuable complementary tool in fertility care, even if they do not directly improve emotional distress or quality of life in the short term.

While offering the MoMiFer-app seemed to enhance mindfulness skills based on self-report questionnaires, no similar effect was observed when assessing mindfulness skills through ESM. A possible explanation is that ESM measured only two aspects of mindfulness skills, namely present-moment awareness and decentering (Van der Gucht et al., 2019) with a limited number of items. In contrast, the CHIME retrospective questionnaire provided a more comprehensive assessment of multiple aspects of mindfulness skills (Cladder-Micus et al., 2019). Additionally, retrospective bias might also explain this discrepancy. Furthermore, compliance with ESM was low and decreased over time. This low compliance with ESM may be due to several factors, including the demanding nature of the method, and the ESM configuration in this study, such as non-tailored prompts and a short notification validity period (Van Berkel, Ferreira & Kostakos, 2017). Future research should focus on increasing compliance to ESM by for example offering a fitting compensation or making the data collection intrinsically rewarding for participants (Van Berkel, Ferreira & Kostakos, 2017).

### Strengths and limitations of the MoMiFer-RCT

To the best of our knowledge, the MoMiFer-RCT is the first study that investigated the impact of a stand-alone mobile mindfulness app on mental health outcomes for people with infertility. An important strength of this pilot RCT was the combination of self-report questionnaires, with in the moment measurements through ESM (Csikszentmihalyi & Larson, 2014, pp. 35–54) within a repeated measures design. Second, the development of the stand-alone mobile mindfulness app was guided by the steps of the medical research council (MRC), which included involvement of patients with infertility, and health care professionals (Boedt et al., 2021; Craig et al., 2008; MRC, 2000). Given the smartphone-delivered, stand-alone character of the app, the MoMiFer-app could reach a broad audience in an accessible and cost-efficient way. Furthermore, offering the intervention through a mobile application aligned with fertility patients’ need for low-treshhold and time efficient mental health support in addition to an already time-intensive fertility trajectory (Boedt et al., 2021).

A couple limitations should also be addressed. First, the three participants who used an alternative mindfulness application during the study were retained in the analysis to preserve the sample size. However, this may have introduced potential bias into the results. Furthermore, all participants were Caucasian and most had a higher level of education (i.e., bachelor or master’s degree), limiting the generalizability of results. Another limitation of this study is that it did not assess the occurrence of stressors, such as minor daily stressors or major stressful life events like setbacks in fertility treatment, which could have influenced the findings. The presence of these stressors during the study period might have affected participants’ well-being and could explain the lack of significant effects on primary outcomes. Future studies should consider assessing the occurrence of stressors that might influence study outcomes.

Despite our promising aim to include men and couples in this clinical trial, only 11 heterosexual couples participated (15%). No men participated independently. Consequently, analysis was performed solely at the individual level. However, fertility-related challenges are a shared experience. Future research should focus on effectively reaching men and couples, examining barriers in this context, and potentially including features aimed at fostering partner involvement (Martins et al., 2016).

## Conclusion

In conclusion, the MoMiFer-RCT revealed that a mobile mindfulness app significantly improved self-compassion and mindfulness skills in people experiencing infertility. Nevertheless, no significant improvements were observed in emotional distress, fertility-related quality of life, or repetitive negative thinking. Enhanced mindfulness skills and self-compassion may indirectly benefit people experiencing infertility their coping with infertility-related stress by fostering emotional regulation and reducing self-criticism. This suggests that mindfulness-based interventions can be valuable in fertility care, even if they do not directly improve emotional distress or quality of life in the short term. The trial’s timing during the COVID-19 pandemic and low app usage may have influenced the outcomes. Further research on potential stressors and ways to increase user adherence is needed to better understand the app’s impact.

## Data Accessibility Statement

Questionnaire data was collected through the online survey platform Qualtrics according to GCP. The data from the stand-alone mobile mindfulness application can be retrieved from the secured website of MoMiFer. Regarding data sharing, the International Committee of Medical Journal Editors recommendations were be followed. Individual deidentified participant data will be shared. Individual participant data that underlie the results reported in our article, after deidentification (text, tables, figures, and appendices) will be shared. Data will become available from 9–36 months after the publication of the RCT-results. Data sharing with research groups interested in performing further data-analysis is stimulated.

## Additional File

The additional file for this article can be found as follows:

10.5334/pb.1375.s1 Supplementary material. Supplement 1 to 4.
