# Supplementary material for: Stand-Alone Mobile Mindfulness App for People Experiencing Infertility: A Pilot Randomised Controlled Trial (MoMiFer-RCT)
Source: Psychol Belg. 2025 Oct 6;65(1):202–16. doi: 10.5334/pb.1375 (PMC12513356; doi:10.5334/pb.1375)
Supplement: Supplementary material. — Supplement 1 to 4. [file pb-65-1-1375-s1.pdf]

Title

Impact of a Stand-Alone Mobile Mindfulness App in People Experiencing Infertility: a Pilot Randomised Controlled Trial (MoMiFer-RCT)

Authors

Tessy Boedt[1,2], Nele Willaert[2,3], Sharon Lie Fong[4,5], Eline Dancet[4,5], Filip Raes[3], Katleen Van der Gucht[2,3,6]

Affiliations

1 Department of Chronic Diseases and Metabolism, KU Leuven, Leuven, Belgium

2 Tilburg School of Social and Behavioral Sciences, Tilburg University, Tilburg, The Netherlands

3 Faculty of Psychology and Educational Sciences, KU Leuven, Leuven, Belgium

4 Leuven University Fertility Centre, University Hospitals Leuven, Leuven, Belgium

5 Department of Development and Regeneration, KU Leuven, Leuven, Belgium

6 Department of Rehabilitation Sciences, Neuromodulation Laboratory, Biomedical Sciences Group, KU Leuven, Leuven, Belgium

Corresponding author

Tessy Boedt

tessyboedt@gmail.com

Tel +32 (0)498 82 80 66

Orcid

Tessy Boedt: 0000-0001-5417-4258

Nele Willaert: 0000-0003-3281-6090

Sharon Lie Fong: 0000-0002-1618-7573

Eline Dancet: 0000-0003-0356-930X

Filip Raes: 0000-0003-2770-2806

Katleen Van der Gucht: 0000-0003-0206-4131

Supplement 1. CONSORT checklist

Section/topic	No	CONSORT 2025 checklist item description	Reported on page no.
**Title and abstract**			
Title and structured abstract	1a	Identification as a randomised trial	1
	1b	Structured summary of the trial design, methods, results, and conclusions	1
**Open science**			
Trial registration	2	Name of trial registry, identifying number (with URL) and date of registration	4
Protocol and statistical analysis plan	3	Where the trial protocol and statistical analysis plan can be accessed	4
Data sharing	4	Where and how the individual de-identified participant data (including data dictionary), statistical code and any other materials can be accessed	22
Funding and conflicts of interest	5a	Sources of funding and other support (eg, supply of drugs), and role of funders in the design, conduct, analysis and reporting of the trial	TP
	5b	Financial and other conflicts of interest of the manuscript authors	TP
**Introduction**			
Background and rationale	6	Scientific background and rationale	2
Objectives	7	Specific objectives related to benefits and harms	2
**Methods**			
Patient and public involvement	8	Details of patient or public involvement in the design, conduct and reporting of the trial	NA
Trial design	9	Description of trial design including type of trial (eg, parallel group, crossover), allocation ratio, and framework (eg, superiority, equivalence, non-inferiority, exploratory)	4
Changes to trial protocol	10	Important changes to the trial after it commenced including any outcomes or analyses that were not prespecified, with reason	NA
Trial setting	11	Settings (eg, community, hospital) and locations (eg, countries, sites) where the trial was conducted	4
Eligibility criteria	12a	Eligibility criteria for participants	4
	12b	If applicable, eligibility criteria for sites and for individuals delivering the interventions (eg, surgeons, physiotherapists)	NA
Intervention and comparator	13	Intervention and comparator with sufficient details to allow replication. If relevant, where additional materials describing the intervention and comparator (eg, intervention manual) can be accessed	6
Outcomes	14	Prespecified primary and secondary outcomes, including the specific measurement variable (eg, systolic blood pressure), analysis metric (eg, change from baseline, final value, time to event), method of aggregation (eg, median, proportion), and time point for each outcome	7

Harms	15	How harms were defined and assessed (eg, systematically, non-systematically)	NA
Sample size	16a	How sample size was determined, including all assumptions supporting the sample size calculation	7
	16b	Explanation of any interim analyses and stopping guidelines	NA
Randomisation:			
Sequence generation	17a	Who generated the random allocation sequence and the method used	5
	17b	Type of randomisation and details of any restriction (eg, stratification, blocking and block size)	5
			**Reported on page no.**
Allocation concealment mechanism	18	Mechanism used to implement the random allocation sequence (eg, central computer/telephone; sequentially numbered, opaque, sealed containers), describing any steps to conceal the sequence until interventions were assigned	5
Implementation	19	Whether the personnel who enrolled and those who assigned participants to the interventions had access to the random allocation sequence	5
Blinding	20a	Who was blinded after assignment to interventions (eg, participants, care providers, outcome assessors, data analysts)	5
	20b	If blinded, how blinding was achieved and description of the similarity of interventions	5
Statistical methods	21a	Statistical methods used to compare groups for primary and secondary outcomes, including harms	11
	21b	Definition of who is included in each analysis (eg, all randomised participants), and in which group	11
	21c	How missing data were handled in the analysis	11
	21d	Methods for any additional analyses (eg, subgroup and sensitivity analyses), distinguishing prespecified from post hoc	NA
**Results**			
Participant flow, including flow diagram	22a	For each group, the numbers of participants who were randomly assigned, received intended intervention, and were analysed for the primary outcome	12
	22b	For each group, losses and exclusions after randomisation, together with reasons	12
Recruitment	23a	Dates defining the periods of recruitment and follow-up for outcomes of benefits and harms	7,12
	23b	If relevant, why the trial ended or was stopped	NA
Intervention and comparator delivery	24a	Intervention and comparator as they were actually administered (eg, where appropriate, who delivered the intervention/comparator, how participants adhered, whether they were delivered as intended (fidelity))	12
	24b	Concomitant care received during the trial for each group	12
Baseline data	25	A table showing baseline demographic and clinical characteristics for each group	13
Numbers analysed, outcomes and estimation	26	For each primary and secondary outcome, by group:<br>● the number of participants included in the analysis<br>● the number of participants with available data at the outcome time point<br>● result for each group, and the estimated effect size and its precision (such as 95% confidence interval)<br>● for binary outcomes, presentation of both absolute and relative effect size	13

Harms	27	All harms or unintended events in each group	14-17
Ancillary analyses	28	Any other analyses performed, including subgroup and sensitivity analyses, distinguishing pre-specified from post hoc	14-17
**Discussion**			
Interpretation	29	Interpretation consistent with results, balancing benefits and harms, and considering other relevant evidence	18
Limitations	30	Trial limitations, addressing sources of potential bias, imprecision, generalisability, and, if relevant, multiplicity of analyses	20

Citation: Hopewell S, Chan AW, Collins GS, Hróbjartsson A, Moher D, Schulz KF, et al. CONSORT 2025 Statement: updated guideline for reporting randomised trials. BMJ. 2025; 388:e081123. https://dx.doi.org/10.1136/bmj-2024-081123
© 2025 Hopewell et al. This is an Open Access article distributed under the terms of the Creative Commons Attribution License (https://creativecommons.org/licenses/by/4.0/), which permits unrestricted use, distribution, and reproduction in any medium, provided the original work is properly cited.

*We strongly recommend reading this statement in conjunction with the CONSORT 2025 Explanation and Elaboration and/or the CONSORT 2025 Expanded Checklist for important clarifications on all the items. We also recommend reading relevant CONSORT extensions. See www.consort-spirit.org.

Supplement 2. Experience sampling method (ESM) items

10 items

Participanten antwoorden op een schaal van 0 tot 100; 0 betekent ‘helemaal niet’, 100 betekent ‘zeer veel of heel erg’.

Symptoms of emotional distress items:

1. Hoe angstig voel je je op dit moment? **(anxiety item)**
2. Hoe gestresseerd voel je je op dit moment? **(stress item)**
3. Hoe depressief voel je je op dit moment? **(depression item)**

Mindfulness skills items:

4. Sinds de vorige beep in welke mate was je aan het piekeren over het verleden?
5. Sinds vorige beep in welke mate maakte je je zorgen over de toekomst?
6. Sinds vorige beep in welke mate merkte je op wat er in je lichaam gebeurde?
7. Sinds vorige beep in welke mate was je je bewust van hoe je je voelde?
8. Sinds vorige beep in welke mate was je met je aandacht bij wat je aan het doen was?
9. Sinds vorige beep in welke mate was je in staat gedachten gewoon te observeren zonder erin mee te gaan?
10. Sinds vorige beep in welke mate heb je je overspoeld gevoeld door gedachten?

Supplement 3. Use of MoMiFer app

App engagement

Weekly practice	Days of practice	*n*
	1	2
	2	1
	3	2
	4	1
	5	1
	8	3
	9	3
	11	2
	12	1
< 1 time/week		16
	13	1
	15	2
	16	1
	17	1
	19	1
	20	2
	21	1

	25	1
≥ 1x/week and < 2x/week		10
	27	1
	29	1
	34	1
≥ 2 times/week and < 3 times/week		3
> 3 times/week (1)	55	1

Mindfulness practice frequency in the intervention group

Note. N = 34, missing data for four participants due to a technical error.

Weekly frequency was calculated using a 3-month trial duration (91 days = 13 weeks).

Supplement 4. Participation during Covid-19 pandemic

		N	
Outcome	Baseline	1.5 months	3 months
No pandemic	12	6	2
Pandemic	60	55	54
Response via mail (%) [a]	30 (50)	3 (6)	17 (31)
Total	72	61	56

Participation During the COVID-19 Pandemic

Note. At each assessment phase we looked at whether participants completed the questionnaires during the COVID-19 pandemic.

[a] Response via mail to the open ended research question.
